# Minireview on Glutamine Synthetase Deficiency, an Ultra-Rare Inborn Error of Amino Acid Biosynthesis

**DOI:** 10.3390/biology5040040

**Published:** 2016-10-19

**Authors:** Marta Spodenkiewicz, Carmen Diez-Fernandez, Véronique Rüfenacht, Corinne Gemperle-Britschgi, Johannes Häberle

**Affiliations:** Division of Metabolism and Children’s Research Center, University Children’s Hospital, Steinwiesstr. 75, 8032 Zurich, Switzerland; Marta.Spodenkiewicz@kispi.uzh.ch (M.S.); Carmen.diez@kispi.uzh.ch (C.D.-F.); Veronique.Ruefenacht@kispi.uzh.ch (V.R.); Corinne.gemperle@kispi.uzh.ch (C.G.-B.)

**Keywords:** glutamine synthetase, rare disease, GLUL, epileptic encephalopathy, GS deficiency, glutamine-glutamate-GABA, hyperammonemia, inborn error of metabolism

## Abstract

Glutamine synthetase (GS) is a cytosolic enzyme that produces glutamine, the most abundant free amino acid in the human body. Glutamine is a major substrate for various metabolic pathways, and is thus an important factor for the functioning of many organs; therefore, deficiency of glutamine due to a defect in GS is incompatible with normal life. Mutations in the human *GLUL* gene (encoding for GS) can cause an ultra-rare recessive inborn error of metabolism—congenital glutamine synthetase deficiency. This disease was reported until now in only three unrelated patients, all of whom suffered from neonatal onset severe epileptic encephalopathy. The hallmark of GS deficiency in these patients was decreased levels of glutamine in body fluids, associated with chronic hyperammonemia. This review aims at recapitulating the clinical history of the three known patients with congenital GS deficiency and summarizes the findings from studies done along with the work-up of these patients. It is the aim of this paper to convince the reader that (i) this disorder is possibly underdiagnosed, since decreased concentrations of metabolites do not receive the attention they deserve; and (ii) early detection of GS deficiency may help to improve the outcome of patients who could be treated early with metabolites that are lacking in this condition.

## 1. Introduction

Glutamine synthetase (GS) is a cytosolic enzyme that produces glutamine, the most abundant free amino acid in the human body. Glutamine is a major substrate for various metabolic pathways, and is thus an important factor for the functioning of many organs; therefore, deficiency of glutamine due to a defect in GS is incompatible with normal life. In fact, mutations in the gene encoding the mitochondrial glutamine synthetase I (GSI) of *Drosophila melanogaster* resulted in embryo-lethal female sterility [1] and, likewise, GS was found to be essential in early embryogenesis in mice [1]. It was thus interesting to learn that mutations in the human *GLUL* gene (OMIM *138290), encoding for GS, can cause an ultra-rare recessive inborn error of metabolism—congenital glutamine synthetase deficiency (OMIM #610015). This disease was reported until now in only three unrelated patients, all of whom suffered from neonatal onset severe epileptic encephalopathy [2,3,4]. The hallmark of GS deficiency in these patients was decreased levels of glutamine in body fluids, associated with chronic hyperammonemia. Although the number of patients with this condition is very small, many lessons could be learned from an investigation of their cases. This review aims at recapitulating the clinical history of the three known patients with congenital GS deficiency and summarizes the findings from studies done along with the work-up of these patients. It is the aim of this paper to convince the reader that (i) this disorder is possibly underdiagnosed, since decreased concentrations of metabolites do not receive the attention they deserve; and (ii) early detection of GS deficiency may help to improve the outcome of patients who could be early treated with metabolites that are lacking in this condition.

## 2. Clinical Presentation of Inherited Glutamine Synthetase Deficiency

Congenital glutamine synthetase deficiency is an ultra-rare disease, hitherto reported in only three unrelated patients since its first description in 2005 [2,3,4]. All of the patients were offspring of consanguineous parents. Two patients had Turkish origin (denoted here as patient 1 and 2, Table 1); the third patient was from Sudan (denoted as patient 3, Table 1). Although the survival time was different among the three, the main clinical features were similar.

The initial phenotype was characterized by severe neonatal encephalopathy and by brain malformations in patients 1 and 2, who underwent magnetic resonance imaging (MRI) of their brain as neonates. These two patients were already severely neurologically compromised at birth, and rapidly went into multiple organ failure. Patient 1 died on day 2 of life, while patient 2 lived for four weeks before succumbing to multiple organ failure. In addition to the encephalopathy, this second patient suffered from a severe gastrointestinal disorder with voluminous diarrhea during the first weeks. During the course of her short life (and probably due to systemic glutamine deficiency), she developed a generalized blistering erythema described on histologic examination as a necrolytic migratory erythema [3]. The brain MRI of both of these patients showed general atrophy, marked malformations, abnormal gyration, and white matter lesions. The post-mortem examination of patient 1 revealed a small brain (weight 202 g, while 335 g was expected for the respective gestational age).

In the third patient (patient 3), the presentation and clinical course differed in comparison to the other patients. At birth, Apgar scores were 9 and 10 at 1 and 5 min, and his clinical examinations were all normal, apart from the neurological testing that showed generalized muscular hypotonia, lower limb hyperreflexia, clonus, and staring of the eyes lasting for 2–3 min. At the age of 13 days, he developed drug-resistant generalized tonic–clonic seizures, and his further neurological development was markedly delayed. Brain MRI at the age of 11 and 28 months showed mild to moderate brain atrophy and hypomyelination of the white matter. He had multiple hospital admissions due to uncontrolled seizures, frequent apnea, and recurrent upper and lower airway infections. At 11 months of age, attacks of seizures with bradycardia and desaturation necessitated mechanical ventilation and later a tracheostomy. Furthermore, at 38 months of age, this patient suffered from a single short episode of necrolytic erythema lasting only few days during a period with very low blood glutamine levels [4]. This patient received a trial of enteral glutamine supplementation (described below) [5] and finally died at six years of age from an acute respiratory decompensation.

## 3. Biochemical Presentation

### 3.1. Physiology and Pathophysiology

Glutamine—the most abundant free amino acid in the human body—plays a major role in essential metabolic pathways such as nitrogen metabolism, ammonia detoxification, acid–base homeostasis, osmotic regulation, and cell signaling and proliferation [6,7,8,9,10].

The diagnosis of GS deficiency is suggested if decreased levels of glutamine in any body fluid are found. The three patients described here were diagnosed by severely decreased glutamine levels in plasma and cerebrospinal fluid (CSF). Interestingly, however, in patient 3, the levels of glutamine in plasma and CSF were normal when first investigated in early infancy, but decreased during the course of the disease [4].

Glutamine is a precursor for neurotransmitters and a substrate for immune cells [6,11,12,13]. Moreover, it is a precursor for glucose, purines, pyrimidines, adenosine monophosphate, and nicotinamide adenine dinucleotide (NAD^+^) [9,14,15,16]. It is also a substrate for transamination reactions required for the production of α-ketoglutarate, closure of the methionine salvage pathway, and salvage of α-keto acids [17].

The only way to produce glutamine in the mammalian organism is by endogenous condensation of ammonia with glutamate [14], an ATP-dependent reaction catalyzed by the GS enzyme [18]. Glutamine from nutritional sources does not contribute to the systemic glutamine pool in healthy individuals, as it is mainly metabolized directly upon absorption in the enterocytes of the small intestine [19,20]. In GS deficiency, the small amount of glutamine that is still produced by the mutant GS protein is probably immediately consumed by several metabolic pathways, resulting in chronic systemic glutamine depletion. Medical conditions with pronounced protein catabolism (e.g., any life-threatening illness, severe inflammation, or burns) can also lead to reduction of the systemic glutamine pool [9,21]. However, glutamine levels reported in such cases remain higher than those seen in inherited GS deficiency [22].

### 3.2. Relation of Glutamine and Hyperammonemia

Intestinal degradation of protein from nutritional sources and the production of ammonia by bacteria in the large intestine are main sources of the excess nitrogenous compounds that undergo detoxification in the liver. As a consequence, the ammonia concentration in the portal vein is significantly higher than in venous or arterial blood. The urea cycle and the action of GS are the two main ammonia detoxifying systems, operating anatomically and functionally in sequence: in periportal hepatocytes, the majority of the excess ammonia is incorporated into urea, while a much smaller proportion that escapes the urea cycle is cleared in the perivenous hepatocytes, where it is used for glutamine synthesis [23,24,25]. Accordingly, the *K*_m_ for ammonia is 1–2 mM in urea cycle enzymes but 0.3 mM in GS, underlining that GS provides a high affinity but low capacity ammonia removal in contrast to the high capacity but low affinity system of the urea cycle [24]. Nevertheless, for a complete ammonia detoxification, both systems are required, and the urea cycle alone is not sufficiently effective. The critical role of GS in ammonia detoxification was confirmed by the rise of ammonia concentration in the hepatic vein as well as in the brain after the inhibition of GS by methionine sulfoximine (MSO, an inhibitor of the GS enzyme) in rats [26,27,28]. In line with this, a liver-specific GS knock-out (KO) mouse model was found to develop hyperammonemia [29]. Not surprisingly, in patients with GS deficiency, the decreased levels of plasma glutamine were accompanied by hyperammonemia. In fact, hyperammonemia in these patients results from the insufficient capacity of the perivenous cells to incorporate ammonia into glutamine [3,30]. Patient 2 had moderate hyperammonemia, and patient 3 showed chronic hyperammonemia during his entire life (closely monitored in the first 43 months) [4]. Thus, these two characteristics—low glutamine levels and hyperammonemia—are the main biochemical signs found in patients with GS deficiency (details in Table 1).

### 3.3. Glutamine Synthetase Activity and Expression

**Figure 1:**
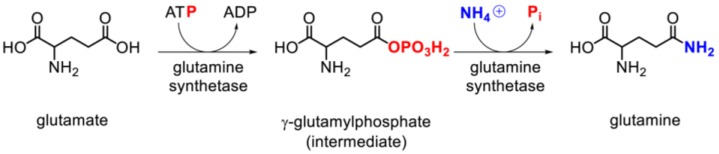
The reaction of glutamine synthetase. Schematic representation of the two-step reaction catalyzed by glutamine synthetase to form glutamine using glutamate, ATP, and ammonia as substrates. The phosphate originating from ATP is depicted in red, ammonium in blue.

GS catalyzes the formation of glutamine in a two-step reaction: Glutamate + ATP → γ-glutamyl phosphate + NH_4_^+^ → glutamine + ADP + Pi [18,24] (Figure 1). The rate of the enzymatic reaction depends on cofactor availability (divalent metal ions), substrate supply, and GS expression levels and activity [3].

In two patients (patients 1 and 2), GS activity was strongly reduced, as measured in immortalized lymphocytes from patient 1 and in COS7 cells transfected with the mutated GS from patient 2 [2,31]. Investigations on fibroblasts from patients 1 and 2 were not possible, since they failed to grow (other GS-deficient fibroblasts required a high-glutamine-containing medium to grow). The longer survival of patient 3 could be related to the higher levels of glutamine synthesis as compared to the other patients (Table 1), suggesting that a slightly higher enzymatic activity of GS was retained in this specific patient. Hence, higher glutamine levels achieved by supplementation can be beneficial. A significant increase of the levels of GS expression was observed in immortalized lymphocytes from patient 1 or in fibroblasts from patient 3 (only for this patient, skin cells survived in a standard medium). This likely reflects an up-regulation mechanism to compensate the low levels of glutamine caused by the diminished enzymatic activity of the mutant GS [2]. This up-regulation could not fully compensate the glutamine deficiency in patients, but may have allowed some pre- and postnatal development, since a complete loss of GS function would not be compatible with survival [1,32,33,34].

GS activity can be compromised not only by inherited defects, but also in a secondary way by posttranslational modifications, such as tyrosine nitration. Excessive ammonia exposure causes the formation of nitric oxide (NO), which interacts with superoxide anions formed in the mitochondria [35,36]. This leads to the creation of highly toxic peroxynitrites, which interact with tyrosine residues of GS [37,38]. In vivo experiments with inducible GS tyrosine nitration in rat hepatocytes showed a decrease of 20% in GS expression and 40%–50% in its activity [39]. Thus, GS tyrosine nitration, observed as a result of chronic hyperammonemia [39,40,41], would explain the decreasing plasma glutamine levels observed in a single patient with GS deficiency (patient 3) [4,36,39].

In rat brain, GS activity can be reduced by NO in astrocytes [42]. In contrast, increased glutamate—by blocking *N*-methyl-d-aspartate (NMDA) receptors or inhibition of NO synthesis (by blocking NO synthase) in vivo—caused an increase of the GS activity and glutamine content in the rat brain [42,43]. Other posttranslational modifications currently studied (such as lysine acetylation of GS) may contribute to the regulation of GS expression. In this case, high glutamine concentrations would cause conformational changes in the protein, exposing the N-terminal domain to acetylation (at lysine residues 11 and 14) to create a signal triggering the enzyme into the degradation pathway [44].

GS function is also regulated by many other factors, including β-catenin activation [45,46], hormonal regulation [47,48,49,50,51], pH variation [9,52], oxidative stress [40,53], glucose deprivation [54], ischemia [55,56], or glutamine concentration in culture medium [57]; however, a detailed description is beyond the scope of this mini-review.

## 4. Molecular Genetics of Glutamine Synthetase Deficiency

The glutamate-ammonia ligase (*GLUL*) gene is highly conserved within living organisms, and is known to be one of the oldest existing genes [58]. In humans, *GLUL* maps to 1q31 [59], and covers 10 kb (Ensembl, ENSG00000135821). One pseudogene and three *GLUL*-like genes have been described, but their function is not fully understood [60]. *GLUL* transcript consists of 1122 bp, it covers 4065 bp (Ensembl, ENST00000417584) comprising six exons encoding a 373 amino-acid polypeptide [2].

Sequence analysis showed three different homozygous missense mutations in *GLUL* exon 6: p.Arg324Cys in patient 1, p.Arg341Cys in patient 2, and p.Arg324Ser in patient 3 (details in Table 1). Parental heterozygosity suggested an autosomal recessive mode of inheritance [2].

Another case of suspected GS deficiency was recently reported to our laboratory in a five year old Russian boy with a severe epileptic encephalopathy progressing since the age of three months. By whole exome sequencing (Centogene, Rostock, Germany), two probably damaging mutations—c.584C > A (p.Ala195Asp) and c.956G > A (p.Arg319His)—were found. However, only the father was a carrier of one of the mutations, but not the mother. Besides, the patient had normal glutamine levels according to tandem mass-spectrometry analysis using dried blood spots. It was therefore necessary to confirm the phase of the variants (*cis* or *trans*), since a double-mutated allele in combination with a wild-type allele was suspected. Thus, in our lab we performed allele-specific long-range polymerase chain reaction (PCR) and Sanger sequencing of *GLUL* in this family. The results suggested the presence of the mutations on the same allele, so the diagnosis of a GS deficiency could not be made in this patient. However, since the two variants are probably disease-causing (see below), they are included in this list of known *GLUL* mutations, and in the structural considerations below.

## 5. Structure of Glutamine Synthetase and Rationalization of the Clinical Mutations

**Figure 2:**
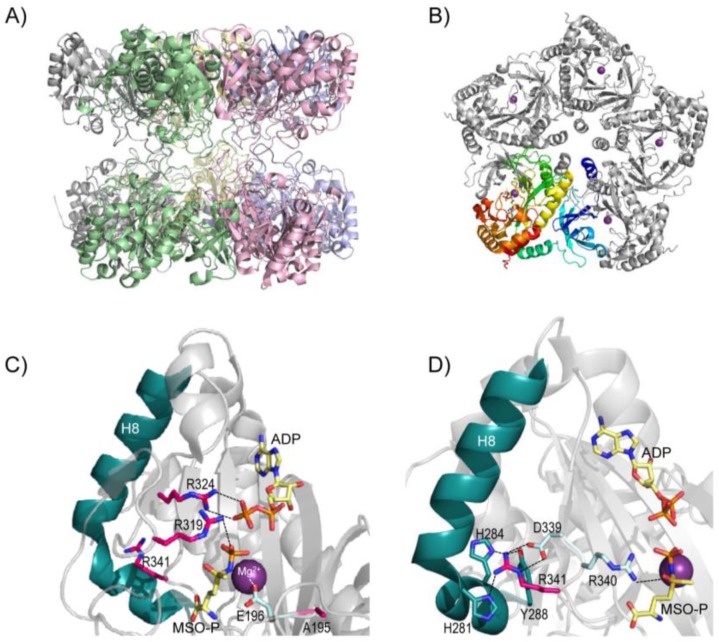
Representation of the crystal structure of human GS (PDB file 2QC8) with the damaging mutations described to-date. (**A**) Human GS decamer showing the two pentameric rings stacking to each other. Each monomer is depicted in a different color. (**B**) Representation of one human GS pentamer with the n1 magnesium ion bound to each monomer (purple spheres). One of the subunits is colored from the N-terminus (blue) to the C-terminus domain (red). (**C**,**D**) Close-up views of the catalytic site of GS, including bound ADP (in sticks), magnesium (purple sphere), and methionine sulfoximine phosphate (MSO-P) (replacing the glutamate). Oxygen atoms are in red, nitrogen atoms in blue, phosphorus atoms in orange, and sulfur atoms in dark yellow. Residues harboring mutations described here are represented as pink sticks. Other highlighted residues are represented as cyan or light blue sticks. Dotted lines represent salt bridges or atom interactions. This figure was adapted from [61,63].

Human GS is a homodecameric protein composed of two pentameric rings stacked to each other (Figure 2A,B). A bifunnel-shaped catalytic site is formed at each interface between two adjacent monomers, adding up to a total of ten catalytic sites per molecule [61]. The association-dissociation process of the monomers of the GS enzyme (as analyzed in the ovine brain in vitro) is regulated by the concentration of the GS protein, substrate, and metal ions [62].

Glutamine is formed via a two-step reaction (Figure 1). In the first step, ATP binds GS, leading to a conformational change that enables glutamate binding. Once glutamate is bound, the terminal phosphate group of ATP is transferred to the γ-carboxylate group of glutamate, producing ADP and γ-glutamyl phosphate (GGP). In the second step, an ammonium ion binds, transfers a proton to Asp63, and the ammonia thus formed attacks GGP. This yields an inorganic phosphate and a positively charged intermediate that is stabilized by Glu305 via a salt bridge interaction. Lastly, Glu305 gets protonated, destabilizing the salt bridge and leading to the opening of glutamate binding site and to glutamine release [63].

Two of the clinical mutations known—p.Arg324Cys (in patient 1) and p.Arg324Ser (in patient 3)—affect a highly-conserved residue [64], Arg324. The prediction server PolyPhen gives predictions of “possibly damaging” for both mutations, and the prediction server MutPred gives values of 0.960 and 0.908 (indicating pathogenicity), respectively. In fact, mutant p.Arg324Cys exhibited 12% of the wild-type enzyme activity when measured in immortalized lymphocytes [2], and p.Arg324Ser conserved certain residual activity [5]. This arginine (Arg324), located in each catalytic site, forms an ionic salt bridge with the β-phosphate group of the ADP in the crystal structure (Figure 2C; PDB file 2QC8, [61]), and therefore will probably be involved in the binding of ATP in the reaction of glutamine synthesis. Thus, removal of this arginine will presumably hamper ATP binding, explaining the decreased enzyme activity observed in both mutant forms [2,5]. Besides, since p.Arg324Cys and p.Arg324Ser had increased expression levels in cell culture studies [2,31], these mutants probably lead to lower catalytic efficiency. It is also to be highlighted that, in homozygosis, p.Arg324Cys was fatal in the neonatal period, while p.Arg324Ser was found in a patient still alive at the age of 6 years. In silico modeling of these two mutant forms predicted an attenuated effect of mutant p.Arg324Ser as a result of water-mediated interactions with ATP [63].

Regarding p.Arg341Cys, the prediction servers give calculations of “possibly damaging” (PolyPhen) and 0.920 (MutPred). This mutation affects an arginine conserved in all eukaryotes [64], and its activity measured in transfected COS7 cells was significantly reduced (to ~58% of the wild-type) [2]. In the crystal structure, Arg341 forms a triad with Asp339 and Arg340 (Figure 2D). This last arginine (Arg340) makes a hydrogen bond and a salt bridge with the sulfoximine group of methionine sulfoximine phosphate (MSO-P) (Figure 2D), a molecule used in the crystal structure to simulate the positioning of glutamate. In addition, the guanidine group of Arg341, pointing away from the catalytic center, establishes hydrogen bonds with His281, His284, and Tyr288, all localized in helix 8 (H8) (Figure 2D). Upon ATP binding (in the first step of the catalysis), this helix undergoes a conformational change, leading to the closure of the GS catalytic site, which has been proven to be essential for glutamate binding [61]. For these two reasons, it was suggested that the removal of Arg341 may hamper glutamate binding to GS [63], in line with the observed decreased activity of the mutant form p.Arg341Cys [2].

Next, the novel mutation p.Arg319His was predicted to be probably damaging (PolyPhen) and received a value of 0.929 (indicating pathogenicity) by MutPred. Arg319 is a conserved arginine in all known species [61], highlighting its essential role. In the crystal structure, this arginine belongs to the secondary elements that undergo a major conformational change near the active site upon substrate binding (amino acids 311–337) (PDB file 2QC8) [61]. In fact, Arg319 binds the phosphate group of MSO-P (Figure 2C) [61], which indicates the direct involvement of this arginine in glutamate binding. p.Arg319His was found in the same allele together with p.Ala195Asp in the child with suspicion of GS deficiency (not confirmed, as described above). For this second mutation, the prediction servers gave calculations of “possibly pathogenic” (PolyPhen) and 0.722 (MutPred). Ala195, in spite of not binding directly any of the substrates, is localized in a loop forming one of the walls of the substrate-binding cavity (Figure 2C) [61]. Its neighboring residue Glu196 is involved in binding one of the three Mn^2+^ ions in the active site (known as n1). In the *apo* structure of GS of *Canis familiaris*, n1 hosts only one divalent cation. Its positive charge serves to compensate for the charges on the three acidic residues involved in catalysis (glutamates 136, 196, and 203) [61]. Thus, the introduction of another negative charge provided by the aspartate (p.Ala195Asp) into this complex network of charges will lead to an imbalance of charges, probably displacing these key glutamates, thus hampering catalysis. In conclusion, these two mutations found in the same allele in this child will probably exhibit additive effects, having an even greater impact on the mutant enzyme. However, this child still carried a wild-type allele, which ensures him normal levels of systemic glutamine.

## 6. Pathophysiology of Glutamine Synthetase Deficiency

**Figure 3:**
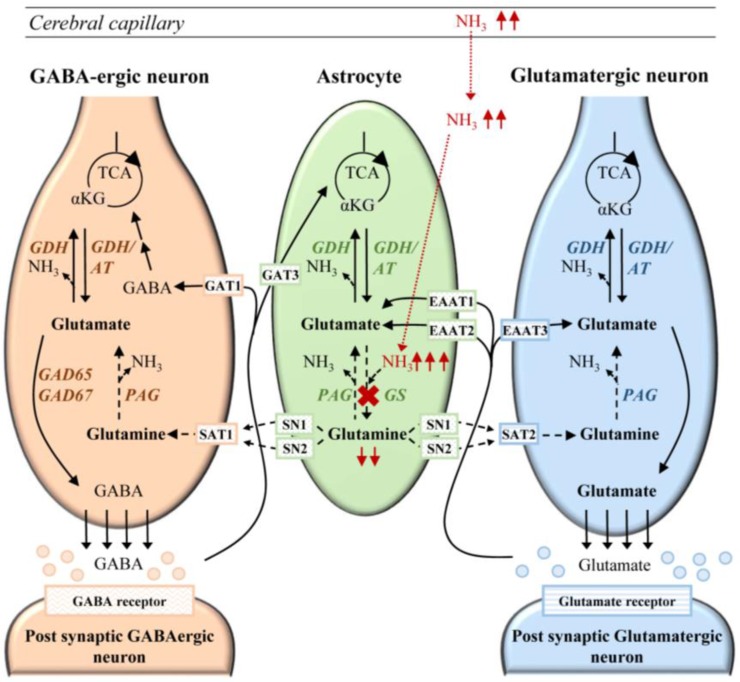
Schematic representation of the hypothetical pathophysiology of GS deficiency in the brain. In GS deficiency, ammonia is elevated in the body circulation and diffuses via the blood–brain-barrier into the brain (indicated by a red dotted line). The defect in the GS reaction results in decreased ammonia detoxification, an excess of ammonia (red upward arrows), and lack of glutamine (red downward arrows), leading to a failure to replenish the glutamine-glutamate-GABA cycle. Intermediates of the TCA cycle: αKG: α-ketoglutarate; Enzymes: GDH, glutamate dehydrogenase; AT, aminotransferases; GS, glutamine synthetase; GAD65, glutamate decarboxylase 65; GAD67, glutamate decarboxylase 67; PAG, phosphate-activated glutaminase; Transporters: GAT1, GABA transporter 1; GAT3, GABA transporter 3; SAT1, system A transporter 1; SAT2, system A transporter 2; SN1, system N transporter 1; EAAT1, excitatory amino acid transporter 1; EAAT2, excitatory amino acid transporter 2; EAAT3, excitatory amino acid transporter 3; Neurotransmitter: GABA, gamma-aminobutyric acid.

Despite its expression in many organs, the main clinical pathology in GS deficiency is related to the central nervous system, where GS is mainly expressed in both astrocytes and oligodendrocytes [65,66,67], but also in Müller cells of the retina [68], ependymal cells, and in nitrergic neurons [69]. GS plays a key role in (i) protecting the neurons from excitotoxicity by capturing ammonia and glutamate in the glial cells; and (ii) supplying glutamine for glutamate and GABA synthesis in the glutamine-glutamate-GABA cycle, regulating the excitatory and inhibitory synaptic transmission of neurons (Figure 3) [6,70,71]. Here, we will only briefly discuss the pathophysiology in relation to GS deficiency, but the reader is referred to more dedicated work for complete insight.

### 6.1. Cerebral Ammonia Toxicity in GS Deficiency

In the brain, ammonia is produced by the reactions of glutaminase and glutamine dehydrogenase [6]. In addition, ammonia from the body circulation can diffuse across the blood-brain barrier, entering astrocytes, where it is normally rapidly detoxified by incorporation into glutamine (Figure 3) [15,47,72]. The GS reaction, together with phosphate-activated glutaminase (PAG), ensures the transfer and regulation of ammonia between neurons and astrocytes [73]. In fact, the reaction driven by GS (and obviously impaired in an inherited defect of this enzyme) is the main mechanism in the brain for removing ammonia [6,26,74]. Any excess of ammonia is toxic for the cerebral cells, by multiple mechanisms. This includes direct toxic effects, such as oxidative/nitrosative stress due to disturbance of the NO pathway, creatine deficiency, and inhibition of the tricarboxylic acid cycle (TCA). These toxic effects can lead to secondary mitochondrial failure, and thus, energy deficit. For a complete picture, the reader is referred to the following reviews [38,47,74,75]. Additionally, the similarity between NH_4_^+^ and K^+^ may lead to an increased up-take of ammonium and water, mediated by the Na^+^/K^+^-ATPase and NKCC1, a co-transporter of Na^+^, K^+^, 2 Cl^−^, and water, contributing to cerebral edema formation during hyperammonemia [75]. By impairing astrocyte potassium buffering, hyperammonemia was found to favor depolarization of GABA neurons, leading to the impairment of the cortical inhibitory network and seizures [75,76]. Histopathologic investigations initially performed on rats with hepatic encephalopathy showed RNA oxidation as a result of elevations in ammonia [41,77]. This was further confirmed in liver-specific GS-deficient KO mice. These mice preserved normal glutamine concentrations in the brain, but showed (due to hyperammonemia) high levels of oxidized RNA in Purkinje cells of the cerebellum, in the hippocampus, and the somato-sensory cortex [29].

According to the “Trojan horse” hypothesis, in hyperammonemic conditions, glutamine (the Trojan horse) serves as a carrier of excessive ammonia into the cytoplasm and into the mitochondria of the astrocytes, causing mitochondrial permeability transition (MPT) [78,79,80,81]. This hypothesis was however discussed because a rise of ammonia was not confirmed in magnetic resonance spectroscopy (MRS) studies with [^15^N] in rats in acute hyperammonemic conditions in vivo. Further, the resulting losses of ATP secondary to MPT were not noted in the hyperammonemic brain [28]. Besides, since the increase of cellular ammonia in the Trojan horse hypothesis relies on the increased production of glutamine in the GS reaction, it may not be present in patients with GS deficiency. Still, this possibility has to be considered during glutamine supplementation, and renders careful ammonia monitoring advisable during the initiation of glutamine therapy in GS-deficient patients.

### 6.2. Neurotransmission in Glutamine Synthetase Deficiency

Glutamine is a substrate for glutamate and GABA synthesis in glutaminergic and GABAergic neurons, respectively, and therefore, it contributes to the regulation of the glutamine-glutamate-GABA cycle compartmentalized between neurons and astrocytes (Figure 3) [6,71,72,75,82]. This was confirmed by nuclear MRS using ^13^C glutamine [71,83,84,85] and by immunochemical studies, where inhibition of GS with MSO increased the level of glutamate in glial cells [11,86]. Further, whole brain metabolites analysis in rats showed that inhibition of GS with MSO decreases the whole glutamate brain pool [6].

Since the availability of glutamine is essential for GABA and glutamate synthesis [71], low glutamine levels in GS deficiency may be insufficient to replenish the glutamate and GABA neuronal pools [87]. As reported in patient 3, glutamine levels in the CSF and on MRS of basal ganglia were very low [5], but could be enhanced by glutamine supplementation, likely improving levels of glutamine in the glutamatergic and GABAergic neurons [5]. This was suggested by the finding of increased brain glutamine and glutamate in the treated GS-deficient patient, although CSF and MRS measurements only partially reflect the neurotransmitter pool [88]. The failure to completely correct the biochemical brain phenotype may, however, be due to brain atrophy as a result of neurodegeneration [5].

Several studies have shown that the inhibition of GS as well as hyperammonemia can lead to an elevation of extracellular glutamate levels [82,89,90], which clinically is often associated with seizures through the activation of the NMDA receptor [38]. However, the concentrations of glutamate were not elevated in the plasma or CSF of patients with GS deficiency, and were even slightly decreased on MRS in patient 3 [5]. Nevertheless, a general impairment of astrocyte function due to glutamine deficiency and hyperammonemia in the patients may lead to a failure of the regulation of neuronal homeostasis and disturbance of the excitation-inhibition balance [91]. Astrocyte dysfunction may thus contribute to abnormal neuronal activity and seizures, leading to severely abnormal electroencephalography (EEG) recordings in GS-deficient patients. In line with this, glutamine supplementation could improve the seizure activity in patient 3 [5]. Further, supporting the role of exogenous glutamine administration, selective viral induction of astrocytic gliosis in a hippocampal region of mouse brain led to a failure of the glutamine-glutamate-GABA cycle and reduced inhibitory synaptic current that could be reversed by glutamine [92].

### 6.3. Fetal Development in Glutamine Synthetase Deficiency

GS is already expressed in early fetal stages [87]. Its activity varies during fetal development, but is essential for early embryogenesis, as shown by the lethality of complete KO mice at only day 3.5 of embryonic development [87]. In addition, in the same study, the embryonic mice could be rescued by glutamine supplementation [87].

During pregnancy, the developing fetus utilizes large amounts of glutamine derived from maternal blood or obtained from endogenous placental synthesis [32,93], which may however be insufficient for a GS-deficient fetus. In human fibroblasts, glutamine was proven essential during fetal development for cellular proliferation [34]. Glutamine and glutamate are also important for the developing brain, in the organization of neural differentiation and proliferation [53,94]. An insufficient supply of glutamine during the embryonic period probably favors white matter changes and brain malformations, as observed on the cerebral MRI of the three patients (Table 1). Interestingly, Cre-mediated astrocyte-specific GS-deficient mice showed glutamine deficiency and increased ammonia in the cerebral tissues, but did not show brain malformation at birth [87]. One possible explanation is that the late elimination of the GS—completed at only 18 days of fetal age in this experiment—may have still allowed early brain development. Further underlining the role of glutamine for the glutamine-glutamate-GABA cycle and glial differentiation during the peri- and postnatal period is the fact that mice deficient in glutamine died three days after birth [87,95].

### 6.4. NAD^+^ Depletion in Glutamine Synthetase Deficiency

Due to the key role of GS and glutamine for the human organism, its deficit can have an impact on many metabolic pathways. It has been noticed that two of the patients presented necrolytic erythema associated with very low plasma glutamine levels. The histological lesions of one patient (patient 2) were reminiscent of pellagra, a disease caused by niacin deficiency [3]. Niacin itself is one of the precursors of nicotinamide adenine dinucleotide (NAD^+^) [96,97]. NAD^+^ (and its phosphorylated and reduced forms, NADP^+^, NADH, and NADPH) is a coenzyme involved in vital oxido-reduction reactions found in all living cells [98,99]. We had the opportunity to investigate the availability of NAD^+^ in one patient with inherited GS deficiency (patient 3) to follow-up on the hypothesis of a NAD^+^ deficiency in patients with systemic glutamine deficiency. Unsurprisingly, NAD^+^ was found to be severely lacking in cultured fibroblasts, immortalized peripheral blood stem cells, and in leukocytes both in vitro and in vivo [31]. Consequently, it was speculated that the NAD^+^ deficiency contributed to the global severity of the disease. Indeed, glutamine is a nitrogen provider for NAD^+^ synthesis by NAD^+^ synthetase; thus, lack of glutamine may contribute to NAD^+^ deficiency found in patients [100]. Furthermore, this observation could open the possibility of a novel therapeutic intervention to alleviate the devastating phenotype in GS deficiency. Exogenous supplementation with either glutamine or NAD^+^ precursors (nicotinamide or nicotinate) in fibroblasts in vitro normalized the intracellular NAD^+^ concentration [31]. The same was observed in vivo when the GS-deficient patient received glutamine over four weeks, resulting in a marked increase in NAD^+^ levels in leukocytes [31].

## 7. Secondary Glutamine Synthetase Deficiency

Secondary deficiency of GS function has been associated with a broad range of human diseases. As underlying cause, a disturbance of nitrogen metabolism or impaired GS activity and expression were hypothesized to contribute to the development of different neurological diseases such as Alzheimer’s, schizophrenia, and mesial temporal lobe epilepsy (MTLE, described below). However, all of these probably result from different perturbations of the posttranslational modifications of GS.

In the case of Alzheimer’s disease, it was hypothesized that GS is vulnerable to either age-related oxidation or to the action of β-amyloids (in the frontal cortex), which would lead to a decrease in its activity [101,102]. An increased expression of GS would be associated with increasing levels of Alzheimer-type pathology in the aging brain, as a reaction to the activation of phosphoinositide-3-kinase-FOXO signaling in astrocytes [103,104]. Additionally, the distribution of the GS expression in the cerebral cortex is perturbed, and may be triggered by toxic agents in senile plaques, reduced noradrenergic supply to the cerebral cortex, and increased brain ammonia levels [102]. The resulting secondary GS deficiency progressively leads to incomplete ammonia detoxification and a gradual decline of the glutamine-glutamate-GABA cycle, causing impairment in synaptic connectivity with deficient cognition and memory [102,105].

Secondary GS deficiency in astrocytes of the sclerotic hippocampal formation was suggested to be a possible molecular basis for MTLE. In this case, it is thought that the deficit of the enzyme leads to chronic accumulation of excitotoxic glutamate in astrocytes and the extracellular space, possibly resulting in the perturbation of the glutamine-glutamate-GABA cycle [82].

Decreased GS activity was also reported in patients who underwent orthotopic lung transplantation for various pulmonary diseases [106]. In a retrospective study, 6 of 145 patients developed severe hyperammonemia following lung transplantation [106]. It was discussed that the hyperammonemia was related to hepatic GS deficiency, which could be confirmed by reduced enzymatic activities of 12% and 28% of the mean value of controls in two cases [106,107]. Among all of the patients, levels of plasma ammonia were elevated to a maximum of 5000 µmol/L (normal < 50) [107], which makes it likely that other factors were contributing as well.

Additionally, since glutamine was found to be an energized substrate for numerous types of tumoral cells [108,109], and GS expression levels were related with survival in some patients [110,111], GS could be a possible pharmacological target in some cancers. For more information about glutamine metabolism in cancer, the reader is referred to the following reviews [109,112].

## 8. Management of Patients with Inherited Glutamine Synthetase Deficiency

The management of patients with GS deficiency is not remedial, but supportive, and depending on the symptoms. Focus is on treatment of epilepsy and on supportive measures to allow the best possible development, despite the disease. As this disorder was devastating in all three patients, palliative care should also be considered early in the course of such patients.

In addition, there are specific considerations related to the metabolic phenotype. For instance, dietary interventions have not yet been tested for this disease. One rationale would be to use protein restriction to alleviate chronic hyperammonemia, but the efficiency of this measure should be evaluated.

Since the normalization of glutamine levels was considered to be the prerequisite for improvement of brain function [5], specific enteral glutamine supplementation was applied over a period of four weeks in one GS-deficient patient (patient 3 in Table 1). Glutamine supplementation was already proven safe in healthy volunteers, and had a positive effect in clinical trials—e.g., for brain development in supplemented preterm children [14,113]. The aim of treatment in the GS-deficient patient was to progressively increase the concentration of glutamine while avoiding its adverse effects, namely the potentially damaging consequences of high ammonia levels in CSF (and of high levels of CSF glutamate, described above).

The patient remained clinically stable during the entire trial. He was provided with l-glutamine via a gastrostomy tube or parenteral route (starting with a dose of 17 mg/kg/day and increasing progressively to 1020 mg/kg/day). Enteral glutamine supplementation was sufficient to establish and maintain normal plasma glutamine levels and increased CSF glutamine levels, which were concomitant with an improvement of the patient’s alertness and emotional expressions. EEG recordings were improved in both awake and sleeping states; in the MRS of basal ganglia, brain glutamine concentrations were markedly increased, reflecting a relevant transfer of glutamine into the brain tissues [5]. Ammonia levels in the plasma and CSF did not increase, but remained stable; nevertheless, in case of severely impaired hepatic function, treatment with high dose glutamine is not recommended [114]. Additionally, since high doses of glutamine could yield mitotoxic levels of ammonia through the reaction of PAG in the astrocytes [115] (Figure 3), it is recommended to start with low-dose glutamine supplementation followed by gradual increases of the dose [5].

Glutamine concentrations remained low in comparison to the normal range for the age, but this may indicate an enhanced glutamine utilization by the glutamatergic and GABAergic neurons, as supported by the improvements in background EEG activity and alertness.

Baseline MRI showed increased white matter intensity on T2 weighted images, reduced white matter volume, and atrophic basal ganglia, and did not improve during the four weeks of the trial. The increase of NAD^+^ in leucocytes (described above) also supported the beneficial effects of the treatment at the cellular level [5,31].

These results corroborate that enteral glutamine supplementation increased the supply of glutamine throughout the body. For future patients, glutamine supplementation should be considered as early as possible in order to prevent the devastating natural course of the disease. In addition, NAD^+^ supplementation may also be beneficial, but should be tested prospectively.

## 9. Conclusions

In summary, we report here the clinical course of the three patients diagnosed to date with an inherited defect in GS. This ultra-rare disease is characterized by severe neonatal encephalopathy with decreased levels of glutamine in body fluids, and is associated with chronic hyperammonemia. GS deficiency is an ultra-rare but probably underdiagnosed disorder that may escape diagnosis because decreased plasma and urine glutamine levels may not be noted as reliably as increased levels. Therapeutic intervention in a single patient using glutamine supplementation demonstrated the feasibility of correcting systemic glutamine deficiency without worsening the pre-existing hyperammonemia or provoking toxic CSF glutamate levels. The severity of GS deficiency also seems to be related to decreased NAD^+^ levels, which were detected in the patient’s cells and were normalized upon glutamine supplementation. For future patients, glutamine supplementation should be administered as early as possible in order to prevent the devastating natural course of the disease. Since GS is a key enzyme for many metabolic pathways, its secondary deficiency, resulting from posttranslational modifications, can also contribute to the development of a broad scope of human neurodegenerative and neurological diseases.

## Figures and Tables

**Table 1:** Main clinical features and diagnostic analysis of patients with glutamine synthetase (GS) deficiency.

Variable	Patient 1 [2]	Patient 2 [2]	Patient 3 [4]
**Ethnicity/Gender**	Turkish boy	Turkish girl	Sudanese boy
**Prenatal ultrasonography**	Polyhydramnios, large lateral brain ventricles with a left frontal paraventricular cyst, micromelia	Dilatation of cerebral posterior fossa	Delayed gyration, marked white matter changes, subependymal cysts
**Presentation at birth**	Spontaneously at 35^4/7^ week	Spontaneously at 38^6/7^ week	Term
**Birth weight** **Length** **Head circumfer.**	2220 g (10th percentile) 44 cm (3rd–10th percentile) 34 cm (75th–90th percentile)	2460 g (3rd–10th percentile) 42 cm (<3rd percentile) 28.5 cm (<3rd percentile)	3245 g (50th percentile) 53 cm (90th percentile) 34 cm (50th percentile)
**Clinical course**	Epileptic encephalopathy Multiple organ failure	Epileptic encephalopathy Multiple organ failure Necrolytic erythema Diarrhea	Epileptic encephalopathy Severe developmental delay Necrolytic skin erythema (one episode)
**Onset**	Immediately after birth	Immediately after birth	Day 1
**Life Time**	2 days	28 days	6 years
**Dysmorphic features**	Flat nasal root, low-set ears, flexion contractures at elbows and knees, shortness of limbs	Broad nasal root, low-set ears	None
**EEG**	Generalized seizures, Outbursts of theta waves	Multifocal seizures	Generalized tonic-clonic seizures
**Brain MRI**	Cerebral and cerebellar atrophy, almost complete agyria, immature white matter, multiple paraventricular cysts in frontal and temporal lobes, enlarged lateral ventricles	Small frontal lobe and cerebellum, delayed gyration, marked white matter changes, subependymal cysts	Mild degree of brain atrophy with prominent cortical sulci and sylvian fissures, hypomyelination of white matter, thinning of the corpus callosum
**Glutamine**			
**Serum**	2 μmol/L (normal range: 433–619)	6 μmol/L (normal range: 300–800)	First test: 126 μmol/L, range: 8–354 μmol/L (normal range: 376–709)
**Urine**	Not detectable (normal range: 52–205)	8 μmol/g creatinine (normal range: 640–3230)	
**Cerebrospinal fluid**	11 μmol/L (normal range: 352–885)	12 μmol/L (normal range: 520–1280)	Range: 50–238 μmol/L (normal range: 373–556)
**Glutamate**			
**Serum**	45 μmol/L (normal range: 36–82)	80 μmol/L (normal range: 70–220)	Range: 20–143 μmol/L (normal range: 62–260)
**Urine**	2 mmol/mol creatinine (normal range: 0–30)	34 μmol/g creatinine (normal range: 0–250)	
**Cerebrospinal fluid**	Not detectable (normal range: 1–48)	2 μmol/L (normal range: 2–51)	
**Ammonia**			
**Plasma**		140 μmol/L (normal < 110)	Mean: 94 μmol/L, range: 38–424 μmol/L (normal < 50)
**Mutation**			
	c.970C > T (p.R324C)	c.1021C > T (p.R341C)	c.970C > A (p.R324S)
